# The challenges of COVID‐19 for people with dementia with Lewy bodies and family caregivers

**DOI:** 10.1002/gps.5393

**Published:** 2020-08-18

**Authors:** Alison Killen, Kirsty Olsen, Ian G. McKeith, Alan J. Thomas, John T. O'Brien, Paul Donaghy, John‐Paul Taylor

**Affiliations:** ^1^ Translational and Clinical Research Institute, Biomedical Research Building Newcastle University Newcastle upon Tyne UK; ^2^ Department of Psychiatry, Level E4 University of Cambridge School of Clinical Medicine Cambridge UK

**Key points**

- The physical, cognitive and neuropsychiatric challenges associated with dementia with Lewy bodies make people particularly vulnerable to COVID‐19.
- Adverse effects may also occur from social isolation, the under‐treatment of existing DLB related symptoms/problems and the negative impact on caregivers.
- A vigilant multi‐disciplinary approach is needed to meet the health and psychosocial needs of people with DLB and support family caregivers.

## 1 INTRODUCTION

During the current SARS‐CoV‐2 pandemic dementia has been identified as disproportionally common in adults aged over 65 who develop severe COVID‐19.^1^ Observational data from the International Severe Acute Respiratory and Emerging Infections Consortium also confirms a high prevalence of dementia in older adults hospitalised with COVID‐19.^2^ It is so far unclear whether there is any direct effect of dementia pathologies as dementia is a disease of old age, and thus likely to be associated with a variety of comorbidities, in particular, frailty, which may further exacerbate the risk of severe infection. In addition up to one third of COVID patients have demonstrated neurological sequelae^3^ and there may be both direct (viral infection within the brain, vascular effects) and indirect effects (eg, host immunological response, impact of treatment) of SARS‐CoV‐2 on the brain.^4^ It is therefore possible that SARS‐CoV‐2 infection may accentuate any pre‐existing neurodegenerative disease.

Dementia with Lewy bodies (DLB) represents at least 4.2% of community‐based dementia, and 7.5% of cases in clinical dementia populations.^5^ Under‐diagnosis is however common, meaning the true figure is likely to be higher.^6^ This form of dementia presents with several distinct cognitive, neuropsychiatric, sleep, autonomic and motor symptoms. These include spontaneous alterations in concentration and attention, recurrent well‐formed visual hallucinations, and rapid eye movement (REM) sleep behaviour disorder. Further problems relate to severe autonomic dysfunction (eg, severe constipation, orthostatic hypotension, and urinary difficulties), and spontaneous features of Parkinsonism, including gait impairment.^7^ People with DLB are admitted to hospital more frequently, and utilize inpatient care to a substantially higher degree, than people with Alzheimer's disease (AD) or the general elderly population.^8^ They also have higher and earlier mortality than people with other dementias.^9^, ^10^

The myriad of symptoms associated with DLB results in a complex condition with significant functional disability and a likely vulnerability to COVID‐19. Furthermore, DLB patients may be biased against in treatment decision algorithms that consider multimorbidity, particularly for critical care access. For example within the UK, the current NICE guidance on critical care for COVID‐19 algorithm (https://www.nice.org.uk/guidance/NG159) uses the Clinical Frailty Scale, whereby a higher category of frailty reduces the likelihood of receiving critical care. On a measure of this type, it is probable that people with DLB would score highly as a consequence of both their physical and their cognitive problems.

As the current wave of COVID‐19 recedes and jurisdictions move to staged social distancing, people with DLB may need to remain shielded longer than some other groups due to their susceptibility and age profile. Even where a person with DLB avoids developing COVID‐19 and hospitalisation, adverse outcomes may result from this period of enforced social isolation. People with DLB invariably have their healthcare needs managed by professionals from a wide variety of specialties, including doctors (primary care, psychiatrists, neurologists, geriatricians), nurses, psychologists, occupational therapists, pharmacists, carers, social workers and physiotherapists, in addition to benefiting from the support of charities and community groups. This raises a particular challenge as shielding or social isolation risks at least some healthcare and psychosocial needs remaining unmet.

## 2 PHYSICAL CHALLENGES

One feature of DLB is an increased risk of infection, notably bronchopneumonia.^11^ This is particularly concerning during the current pandemic, where it might exacerbate, or be mistaken for COVID‐19 symptoms. Secondary pneumonias may be more common given that people with DLB often have a pharyngeal‐type dysfunction, which results in a high prevalence of silent aspiration.^12^ This could contribute to diagnostic difficulty especially in conjunction with anosmia, another symptom commonly associated with DLB,^13^ and which is also indicative of COVID‐19.

In common with other dementias, a person with DLB may fail to notice or report symptoms, of, for example, fever, which may indicate COVID‐19 or another infection. Furthermore, coupled with a reduction in the availability of face‐to‐face healthcare provision, infections may progress significantly before detection. Family caregivers are important partners in detecting changes in normal behaviour, which may indicate a developing illness. However, they may need guidance in asking appropriate questions particularly where they are living apart and maintaining contact by phone.

In the UK ‘Telemedicine’ is replacing many face‐to face consultations during the current pandemic and is being viewed as a potential longer‐term alternative for primary care.^14^ In addition to age related hearing/visual impairment, people with DLB may find this form of communication particularly challenging to navigate especially when they are feeling unwell. They have significantly more visual complaints when compared to people with AD, including misjudging objects, difficulty reading, and increased double vision^15^ and thus could struggle to interact with the virtual medium. Language dysfunction is more severe in DLB than other dementias and includes impairments of speech fluency, speech freezing, reduced pitch range and hypophonia.^16^, ^17^ This may lead to problems in both responding to on‐screen questions and being understood. Such challenges need consideration when transforming services to ensure equality of access.

People with DLB are at increased risk of developing delirium if they develop an infection compared with AD.^18^ As infection progresses in severity, treatment becomes more difficult and the risk of delirium and hospitalisation increases. The maintenance of good general hygiene is therefore important to reinforce, for example, where there are continence difficulties, as urinary tract infections can escalate quickly. It is also vital that family caregivers understand the importance of the early assessment of suspected infections by a health care professional. This could include reassurance that perceived pressures on healthcare staff and concerns about overburdening services do not warrant any delay in seeking help. It is important that caregivers inform health care professionals of the DLB diagnosis in any communication. This will highlight to them the need for caution when prescribing anti‐psychotic medication due to the risk of a severe sensitivity syndrome.^19^ Families can assist healthcare professionals to differentiate between fluctuations commonly experienced by their family member and unfamiliar behaviour that may indicate delirium.

Avoiding unnecessary hospital admissions is important, as the change of environment and routine are likely to increase confusion in people with DLB. They may feel particularly isolated if visiting is restricted and they are unable to manage phone contact. It may be advisable for family caregivers to consider in advance whether the person with DLB is sufficiently cognitively aware to express their wishes or preferences regarding any future hospital admission or treatment.

People with DLB may have difficulty in understanding and following instructions regarding the importance of good hand hygiene in reducing the risk of COVID‐19 transmission. Clear, straightforward explanations are key, with frequent repetition as needed. Signs or pictures can be attached above sinks to act as prompts, and caregivers can model good handwashing practice, and give positive reinforcement of successful performance. If the care‐recipient is reluctant, sitting with a bowl of water rather than standing at the sink or incorporating a hand massage may be welcomed. With appropriate signposting, less cognitively impaired people with DLB may be able to access videos demonstrating handwashing techniques.

Other physical symptoms of concern include orthostatic hypotension. This is more common in DLB than other dementias with a greater reduction in systolic blood pressure and a more prolonged period of orthostasis.^20^ This may increase the risk of falls, which is also higher for people with DLB than for other dementias.^21^ This possibility is exacerbated by poor hydration, a potential concern if people are missing the usual prompts from family caregivers. Good hydration can be maintained through reminders by phone, sharing drinks virtually during video calls or alarms set at regular intervals. If there is a risk of falls going undetected, families may need guidance to assess the relative risks of visiting rather than remaining physically distant. Healthcare professionals should be willing to support families in assessing these risks and helping them reach decisions about the most appropriate level of contact. This should take into account both national guidance and the needs of the individual with DLB.

Many people with DLB take short daily walks or participate in low‐level exercise classes offered by gyms or day centres. Regular exercise can interrupt the trajectory of frailty, limitations in mobility, stiffness and sarcopenia, often evident in DLB. Benefits include improvements in gait speed and in the daily activities subsection of the UPDRS.^22^ While the exercise capacity of people with DLB is relatively low in comparison with other cohorts with dementia, any reduction may heighten the falls risk. Maintaining safe home‐based activities, for example, by following appropriate on‐line fitness programmes, in many cases with assistance, may reduce the impact of the lost exercise opportunities and minimise skeletal or muscular pain from reduced mobility.

### 2.1 Cognitive and neuropsychiatric challenges

A lack of meaningful activity adversely affects quality of life in people with dementia,^23^ and people with DLB benefit from cognitively stimulating activities.^24^ They may find the sudden loss of these and the changes to their daily routine necessitated in response to COVID‐19 difficult to comprehend. They may also struggle to understand the need for social isolation, and misinterpret the reason for the lack of visits from family and friends. People with DLB may react with anxiety, anger, stress, or withdrawal in response to worry they perceive in others, or news events about which they have limited understanding. Caregivers can assist by sharing simple non‐threatening messages about COVID‐19, emphasising the temporary nature of the situation, and reassuring the person with DLB of their continued importance to them despite their physical absence. Frequent, brief communication is probably of more value than extended contact where concentration may be difficult to sustain, especially over unfamiliar media platforms.

Non‐pharmacological treatment modalities are recommended to manage behavioural disturbance in people with dementia.^25^, ^26^ This is particularly salient given that delivering face‐to‐face or group non‐pharmacological interventions to reduce agitation, for example, music therapy or sensory stimulation,^27^ may be impossible due to the difficulties in maintaining social distancing. Any resurgence or development of behavioural difficulties may therefore require pharmacological interventions, and caregivers should be alert to the need to discuss concerns and treatment options with health care professionals.

Depression or low mood are common features of DLB^28^ with depression more frequent than in AD.^29^ Depressive symptoms also differ between DLB and AD with apathy and pervasive anhedonia significantly higher in people with DLB.^30^ Apathy often responds to stimulation from social activities and in the absence of these, is important to find alternatives, for example assisting with household tasks, baking or straightforward games. Family caregivers can be encouraged to offer managed choices to reduce any perceived loss of control over how the day is spent. If the person with DLB lives alone and seems disinclined to initiate available activities such as jigsaws, crafts or gardening, caregivers can be encouraged to prompt, *enthuse and encourage* via any accessible communication routes. Resources to enhance activities can be ordered online and depending on the level of impairment, turn‐taking activities may be possible via video calls to maintain engagement. Family caregivers should be advised to contact a health care professional if they detect a deterioration in mood as changes to medication may be warranted.

Reduced social stimulation, a lack of daytime routine, and less physical activity may exacerbate excessive daytime somnolence, a common feature of DLB,^31^ and result in poorer night‐time sleep. Where COVID‐19 guidelines permit, outside exercise can help to regulate this. If the person with DLB is safe to go out alone or accompanied, and compliant with social distancing, a daily walk offers the benefits of both physical exercise and exposure to daylight for melatonin synthesis. Psychological benefits include adding variety to the day, the stimulation of different surroundings and the opportunity to engage at an appropriate distance with passers‐by. Where the weather and circumstances allow, sitting in the garden or at an open front door while maintaining social distancing may also be worthwhile.

Recurrent complex visual hallucinations are a common feature of DLB. They cause significant distress in around 50% of people who experience them, with fear and anger being the most common responses.^32^ They tend to occur in the same location, which is most often within the house, frequently the living room or looking out of the window.^33^ Worsening of visual hallucinations, often coupled with increasing confusion might herald an undiagnosed delirium or infection. However while this possibility should be excluded, hallucinations may also increase in frequency and intensity with more time spent at home and in the rooms where they are typically experienced, particularly where combined with social isolation and reduced sensory input and stimulation from others. People with DLB and caregivers should be alerted to this, and where the hallucinations are causing distress, be given the opportunity to discuss the range of treatment options. Guidelines include both pharmacological and non‐pharmacological strategies^30^ and caregivers can be advised to try distraction, reassurance and bright lighting to try to minimise the occurrence. Rearranging the furniture to alter the location of any items causing misperceptions may help, as can trying to ensure that any corrective lenses, are clean, easily accessible and worn.

Delusions, another common feature of DLB may also increase as a result of anxiety or agitation, perhaps in conjunction with overheard or poorly understood conversations, or news reports that include unfamiliar scenes, such as people wearing personal protective equipment (PPE). Family caregivers can be encouraged to balance their own need to keep informed with protecting the care‐recipient to avoid triggering or reinforcing this behaviour.

Including collateral information from family caregivers in the clinical assessment of neuropsychiatric symptoms is essential to diagnosis and management because people with DLB frequently lack insight regarding the extent of their impairment.^34^ It is important to find ways to facilitate this into telemedicine consultations even when caregivers live physically distant from the care‐recipient. Many people with DLB incorporate what is on the screen into their delusions and the absence of the usual cues of surroundings, equipment or uniforms to make them aware they are talking to a health care professional may cause confusion and present an added challenge.

### 2.2 Challenges for family caregivers

Supporting someone with DLB poses a substantial burden on family caregivers. Compared with AD, caregiver burden in family caregivers of people with DLB is rated 30% higher on the Relative Stress Scale^35^ and a major depressive disorder in the caregiver is significantly more likely.^36^ The COVID‐19 pandemic is likely to create multiple further difficulties.

Physical outcomes may include caregiver fatigue from the increased hours spent supporting the person with DLB with the everyday activities of living, particularly in spousal caregivers who may have age related physical limitations. Additional personal care needs may arise where formal caregivers are unable to visit and routine appointments such as podiatry are unavailable. Many families rely on respite from daycentres or short‐term care home admission to reduce caregiving hours and care demands to a manageable level. The closure of these removes the opportunity for caregivers to ‘recharge’ and requires them to be constantly available. This may be particularly difficult where there is no foreseeable end in sight.

Mental health challenges for caregivers include managing their own anxiety and coping with uncertainty, including over the duration of isolation, whilst containing anxiety in their care‐recipient. Finding ways to accommodate changes to dietary preferences in the face of shortages in essential food items may be stressful. Difficulty obtaining regular prescriptions and appointments such as physiotherapy to maintain mobility may also be concerning for caregivers who are likely to be aware of the detrimental effects of being without these. Feelings of anticipatory grief related to a DLB diagnosis contribute to caregiver burden^37^ and may be heightened by anxiety about their family member contracting COVID‐19. Caregivers with co‐morbidities which put them in a high‐risk group, may worry about becoming ill themselves, and miss the companionship and emotional support of friends and family while socially isolated. They may experience poor sleep as a result of their own anxieties and disturbed sleep in the care‐recipient. Finally caregivers may be disturbed by insensitive media coverage regarding access to critical care, unsure whether or how to undertake advance care planning conversations and nervous about taking responsibility for best interest decisions.

Where the person with DLB is in a care home, caregivers may be distressed by the lack of visiting opportunities and the effect of this on the care‐recipient. They may fear the spread of COVID‐19 from other residents and experience a lack of purpose without their normal visiting routine. Both caregiver and care‐recipient may find the lack of physical touch through being unable to hug or hand hold difficult to manage. Sending in personal items such as photographs, familiar scent worn by the caregiver or playlists may provide some comfort.

It is essential that caregivers of people with DLB feel well supported to enable them to maintain their caregiving role during the COVID‐19 crisis, and health and social care professionals can be sources of both practical and emotional support. Guidelines such as the UK Royal College of Psychiatrists Standards for Memory Services^38^ recommend that caregivers receive a person‐centred service that takes into account their unique and changing personal, psychosocial and physical needs. This includes offering individual time with staff to discuss their own needs. Wellbeing checks instigated by telephone or video call through clinical services such as memory clinics should therefore include caregivers' needs in their assessments. Suggestions to caregivers may include trying to concentrate on those things they can control, that is, making a contingency plan for if they get ill, keeping up good nutrition, and maintaining a daily routine rather than focussing on situations that they cannot control. This may include limiting the time spent watching news updates. They may be encouraged to consider how they have coped with previous challenging situations and whether they can adopt similar techniques again. Practical solutions to pressing problems such as arranging volunteer shoppers and medication deliveries may be as important as offering psychological support.

Detrimental effects for caregivers may also be reduced through maintaining contact with their informal social networks, bearing in mind that where platforms such as video calls are unfamiliar, additional support may be needed. Carers' organisations and faith groups may be beneficial as may opportunities to ‘buddy’ other caregivers and so offer as well as receive support. Accessing DLB specific advice and information from charities such as the Lewy Body Dementia Association (https://lbda.org) and the Lewy Body Society (https://lewybody.org) can also be encouraged.

### 2.3 Challenges for primary care/generalist services

During the urgent restructuring in response to COVID‐19, specialist services in hospital‐based clinics were suspended, and many people with DLB only had access to primary care. Although some services were subsequently reinstated as telephone or video consultations, this highlights an important concern about how primary care teams can access specialist advice and feel equipped to provide proxy specialist care when the need arises. Establishing links between health care professionals in these areas is vital to enable case discussions and requests for urgent reviews to take place, and to facilitate sharing of current practice. This could include distributing details of updated working arrangements and contact details in situations where normal services are undeliverable.

There are other routes by which generalist and primary care services can access information on the best management and care of people with DLB. Many countries employ dementia nurses and they are increasingly taking on proactive roles in the advising on the management of dementia in community settings. In the UK, the first Admiral Nurse specifically for Lewy body dementias has recently been appointed^39^ with a remit that includes supporting health care professionals through telephone advice and the development of training material. A further source of DLB specific information for primary care and generalist services is available via the UK National Institute for Health Research (NIHR) funded Diamond Lewy website https://research.ncl.ac.uk/diamondlewy/. The Diamond Lewy study aimed to improve the diagnosis and management of Lewy body dementia including DLB. Freely downloadable resources include assessments for recognising and diagnosing DLB and summary sheets and detailed recommendations for managing key symptoms. These align with the most recent Consensus Report of the DLB consortium on the diagnosis and management of DLB.^40^, ^41^ They are ideally placed to guide health care professionals having undergone piloting and feasibility testing, and been evaluated as acceptable by clinicians working in general dementia services.^42^

## 3 CONCLUSION

While the effects of COVID‐19 are widespread, it is important to recognise the challenges presented by the significant vulnerabilities associated with DLB. There is a pressing need for research to study the impact of COVID‐19 on this population including ensuring that people with DLB are not excluded from studies because of their age, shielding protocols or their dementia diagnosis. Utilising the person‐centred, problem‐solving approach highlighted here including recognition of the value of a multidisciplinary response can mitigate some of the adverse effects of COVID‐19. This should enable people with DLB and family caregivers to have their health and psychosocial care needs met in the short term, and develop resilience to withstand future COVID‐19 related challenges.

## CONFLICT OF INTEREST

The authors have no conflict of interest to declare.

## References

[1] Atkins J , Masoli J , Delgado J , et al. Pre‐existing comorbidities predicting severe COVID‐19 in older adults in the UKbiobank community cohort. medRxiv; 2020. https://www.rcpsych.ac.uk/docs/default‐source/improving‐care/ccqi/quality‐networks/memory‐clinics‐msnap/msnap‐7th‐edition‐standards‐final.pdf?sfvrsn=b68c8fdb_0. Accessed May 18, 2020.

[2] International Severe Acute Respiratory and Emerging Infections Consortium (ISARIC) . COVID‐19 Report 2020. https://isaric.tghn.org/covid-19-clinical-research-resources/. Accessed May 17, 2020.

[3] Carod‐Artal FJ . Neurological complications of coronavirus and COVID‐19. Rev Neurol. 2020;70(9):311‐322. 32329044 10.33588/rn.7009.2020179

[4] Needham EJ , Chou SH , Coles AJ , Menon DK . Neurological implications of COVID‐19 infections. Neurocrit Care. 2020;32(3):667‐671. 32346843 10.1007/s12028-020-00978-4 PMC7188454

[5] Vann Jones SA , O'Brien JT . The prevalence and incidence of dementia with lewy bodies: a systematic review of population and clinical studies. Psychol Med. 2014;44:673‐683. 23521899 10.1017/S0033291713000494

[6] Kane J , Surendranathan A , Bentley A , et al. Clinical prevalence of lewy body dementia. Alzheimer's Res Ther. 2018;10:19. 29448953 10.1186/s13195-018-0350-6 PMC5815202

[7] Donaghy PC , McKeith IG . The clinical characteristics of dementia with lewy bodies and a consideration of prodromal diagnosis. Alzheimer's Res Ther. 2014;6:46. 25484925 10.1186/alzrt274 PMC4255387

[8] Mueller C , Perera G , Rajkumar A , et al. Hospitalization in people with dementia with lewy bodies: frequency, duration, and cost implications. Alzheimers Dement. 2018;10:143‐152. 10.1016/j.dadm.2017.12.001 PMC5956805 29780862

[9] Price A , Farooq R , Yuan J , et al. Mortality in dementia with lewy bodies compared with Alzheimer's dementia: a retrospective naturalistic cohort study. BMJ Open. 2017;7(11):e017504. 10.1136/bmjopen-2017-017504 PMC5695389 29101136

[10] Mueller C , Soysal P , Rongve A , et al. Survival time and differences between dementia with lewy bodies and Alzheimer's disease following diagnosis: a meta‐analysis of longitudinal studies. Ageing Res Rev. 2019;50:72‐80. 30625375 10.1016/j.arr.2019.01.005

[11] Hanyu H , Sato T , Hirao K , Kanetaka H , Sakurai H , Iwamoto T . Differences in clinical course between dementia with lewy bodies and Alzheimer's disease. Eur J Neurol. 2009;16:212‐221. 19146642 10.1111/j.1468-1331.2008.02388.x

[12] Londos E , Hanxsson O , Alm Hirsch I , Janneskog A , Bülow M , Palmqvist S . Dysphagia in lewy body dementia—a clinical observational study of swallowing function by videofluoroscopic examination. BMC Neurol. 2013;13:140. 24099488 10.1186/1471-2377-13-140 PMC4126015

[13] Beach T , Adler C , Zhang N , et al. Severe hyposmia distinguishes neuropathologically confirmed dementia with lewy bodies from Alzheimer's disease dementia. PLoS One. 2020;15:4. 10.1371/journal.pone.0231720 PMC7176090 32320406

[14] O'Dowd A . Doctors question Hancock's idea of GP video consultations for all. BMJ. 2018;362:K3934. 30217932 10.1136/bmj.k3934

[15] Jefferis JM , Clarke MP , Mosimann UP , et al. The influence of two common dementia types on visual symptoms. Acta Ophthalmol. 2000;91:e159‐e160. 10.1111/j.1755-3768.2012.02544.x 23025337

[16] Chiu P‐Y , Hung G‐U , Wei C‐Y , Tzeng RC , Pai MC . Freezing of speech single questionnaire as a screening tool for cognitive dysfunction in patients with dementia with lewy bodies. Front Aging Neurosci. 2020;12:65. 32410979 10.3389/fnagi.2020.00065 PMC7199820

[17] Ash S , Mcmillan C , Gross R , et al. Impairments of speech fluency in Lewy body spectrum disorder. Brain Lang. 2012;120:290‐302. 22099969 10.1016/j.bandl.2011.09.004 PMC3299896

[18] McKeith IG , Ferman TJ , Thomas AJ , et al. Research criteria for the diagnosis of prodromal dementia with lewy bodies. Neurology. 2020;94:743‐755. 32241955 10.1212/WNL.0000000000009323 PMC7274845

[19] Walker Z , Possin KL , Boeve BF , Aarsland D . Lewy body dementias. Lancet. 2015;386:1683‐1697. 26595642 10.1016/S0140-6736(15)00462-6 PMC5792067

[20] Andersson M , Hansson O , Minthon L , Ballard CG , Londos E . The period of hypotension following orthostatic challenge is prolonged in dementia with lewy bodies. Int J Geriatr Psychiatry. 2008;23:192‐198. 17621385 10.1002/gps.1861

[21] Komatsu T . Fall risk and fracture. Falls in patients with dementia. Clin Calcium. 2013;23:731‐738. 23628687

[22] Inskip M , Mavros Y , Sachdev P , et al. Exercise for individuals with lewy body dementia: a systematic review. PLoS One. 2016;11:E0156520. 27258533 10.1371/journal.pone.0156520 PMC4892610

[23] Phinney A , Chaudhury H , O'Connor D . Doing as much as I can do: the meaning of activity for people with dementia. Aging Ment Health. 2007;11:384‐393. 17612802 10.1080/13607860601086470

[24] McCormick SA , Vatter S , Carter L , et al. Parkinson's‐adapted cognitive stimulation therapy: feasibility and acceptability in lewy body spectrum disorders. J Neurol. 2019;266:1756‐1770. 31161388 10.1007/s00415-019-09329-6 PMC6586694

[25] Connors MH , Quinto L , McKeith I , et al. Non‐pharmacological interventions for lewy body dementia: a systematic review. Psychol Med. 2018;48:1749‐1758. 29143692 10.1017/S0033291717003257 PMC6088773

[26] Banerjee S. The use of antipsychotic medication for people with dementia: Time for action; 2009. https://www.jcpmh.info/wp-content/uploads/time-for-action.pdf. Accessed May 18, 2020.

[27] Livingston G , Kelly L , Lewis‐Holmes E , et al. Non‐pharmacological interventions for agitation in dementia: systematic review of randomised controlled trials. Brit J Psychiat. 2014;205:436‐442. 10.1192/bjp.bp.113.141119 25452601

[28] Yamane Y , Sakai K , Maeda K . Dementia with lewy bodies is associated with higher scores on the geriatric depression scale than is Alzheimer's disease. Psychogeriatrics. 2011;11:157‐165. 21951956 10.1111/j.1479-8301.2011.00368.x

[29] Ballard C , Holmes C , McKeith I , et al. Psychiatric morbidity in dementia with lewy bodies: a prospective clinical and neuropathological comparative study with Alzheimer's disease. Am J Psychiatry. 1999;156:1039‐1045. 10401449 10.1176/ajp.156.7.1039

[30] Chiu P‐Y , Wang C‐W , Tsai C‐T , Li SH , Lin CL , Lai TJ . Depression in dementia with lewy bodies: a comparison with Alzheimer's disease. PLoS One. 2017;12:e0179399. 28617831 10.1371/journal.pone.0179399 PMC5472293

[31] Boddy F , Rowan E , Lett D , et al. Subjectively reported sleep quality and excessive daytime somnolence in Parkinson's disease with and without dementia, dementia with lewy bodies and Alzheimer's disease. Int J Geriatr Psychiatry. 2007;22:529‐535. 17096456 10.1002/gps.1709

[32] O'Brien J , Taylor JP , Ballard C , et al. Visual hallucinations in neurological and ophthalmological disease: pathophysiology and management. J Neurol Neurosurg Psychiatry. 2020;91:512‐519. 32213570 10.1136/jnnp-2019-322702 PMC7231441

[33] Collerton D , Perry E , McKeith I . Why people see things that are not there: a novel perception and attention deficit model for recurrent complex visual hallucinations. Behav Brain Sci. 2005;28:737‐794. 16372931 10.1017/S0140525X05000130

[34] Taylor J‐P , McKeith I , Burn D , et al. New evidence on the management of lewy body dementia. Lancet Neurol. 2020;19:157‐169. 31519472 10.1016/S1474-4422(19)30153-X PMC7017451

[35] Svendsboe E , Terum T , Testad I , et al. Caregiver burden in family carers of people with dementia with lewy bodies and Alzheimer's disease. Int J Geriatr Psychiatry. 2016;31:1075‐1083. 26765199 10.1002/gps.4433

[36] Lowery K , Mynt P , Aisbett J , Dixon T , O'Brien J , Ballard C . Depression in the carers of dementia sufferers: a comparison of the carers of patients suffering from dementia with lewy bodies and the carers of patients with Alzheimer's disease. J Affect Disord. 2000;59:61‐65. 10814772 10.1016/s0165-0327(99)00123-8

[37] Park J , Tolea M , Arcay V , et al. Self‐efficacy and social support for psychological well‐being of family caregivers of care recipients with dementia with lewy bodies, Parkinson's disease dementia, or Alzheimer's disease. Soc Work Ment. 2019;17:253‐278.

[38] Royal College of Psychiatrists . Memory Services National Accreditation Programme Standards for Memory Services. 7th ed; 2020. https://www.rcpsych.ac.uk/docs/default‐source/improving‐care/ccqi/quality‐networks/memory‐clinics‐msnap/msnap‐7th‐edition‐standards‐final.pdf?sfvrsn=b68c8fdb_0. Accessed May 18, 2020.

[39] Lewy Body Society . First Ever Consultant Admiral Nurse for Lewy Body Dementia. https://www.lewybody.org/first-ever-consultant-admiral-nurse-for-lewy-body-dementia/. Accessed June 28, 2020.

[40] Thomas AJ , Taylor J‐P , McKeith I , et al. Revision of assessment toolkits for improving the diagnosis of Lewy body dementia: the DIAMOND Lewy study. Int J Geriatr Psychiatry. 2018;33(10):1293‐1304. 30091150 10.1002/gps.4948 PMC6221009

[41] McKeith IG , Boeve BF , Dickson DW , et al. Diagnosis and management of dementia with Lewy bodies: fourth consensus report of the DLB consortium. Neurol. 2017;89(1):88‐100. 10.1212/WNL.0000000000004058 PMC5496518 28592453

[42] Thomas AJ , Taylor J‐P , McKeith I , et al. Development of assessment toolkits for improving the diagnosis of the Lewy body dementias: feasibility study within the DIAMOND lewy study. Int J Geriatr Psychiatry. 2017;32(12):1280‐1304. 27928840 10.1002/gps.4609 PMC5724510

